# Cell-free biodegradable electroactive scaffold for urinary bladder regeneration

**DOI:** 10.21203/rs.3.rs-3817836/v1

**Published:** 2024-01-29

**Authors:** Guillermo Ameer, Rebecca Keate, Matthew Bury, Maria Mendez-Santos, Andres Gerena, Madeleine Goedegebuure, Jonathan Rivnay, Arun Sharma

## Abstract

Tissue engineering heavily relies on cell-seeded scaffolds to support the complex biological and mechanical requirements of a target organ. However, in addition to safety and efficacy, translation of tissue engineering technology will depend on manufacturability, affordability, and ease of adoption. Therefore, there is a need to develop scalable biomaterial scaffolds with sufficient bioactivity to eliminate the need for exogenous cell seeding. Herein, we describe synthesis, characterization, and implementation of an electroactive biodegradable elastomer for urinary bladder tissue engineering. To create an electrically conductive and mechanically robust scaffold to support bladder tissue regeneration, we developed a phase-compatible functionalization method wherein the hydrophobic conductive polymer poly(3,4-ethylenedioxythiophene) (PEDOT) was polymerized *in situ* within a similarly hydrophobic citrate-based elastomer poly(octamethylene-citrate-co-octanol) (POCO) film. We demonstrate the efficacy of this film as a scaffold for bladder augmentation in athymic rats, comparing PEDOT-POCO scaffolds to mesenchymal stromal cell-seeded POCO scaffolds. PEDOT-POCO recovered bladder function and anatomical structure comparably to the cell-seeded POCO scaffolds and significantly better than non-cell seeded POCO scaffolds. This manuscript reports: (1) a new phase-compatible functionalization method that confers electroactivity to a biodegradable elastic scaffold, and (2) the successful restoration of the anatomy and function of an organ using a cell-free electroactive scaffold.

### Introduction

Since the advent of tissue engineering in the late 1980s, the promise of the field ignited substantial initial excitement, yet its clinical impact remains limited to date. Biomaterial innovation has explored the implementation of soluble and immobilized biochemical cues, biomimetic scaffold architectures, and biomechanical and biophysical enhancements.[1–5] Despite these developments, many factors continue to hinder translational success including high R&D costs, manufacturing challenges, clinical costs, and limited efficacy of most synthetic scaffolds.[6] The rigorous regulatory pathways accompanying new materials also present a significant obstacle, and the inclusion of cells or active biologics, while helpful in regenerating tissue, complicates regulatory processes and user adoption.

The bladder is one of the few organs evaluated in humans that has been engineered *in vitro* to replace or augment function *in vivo* using synthetic scaffolds seeded with cells.[7–14] Bladder tissue regeneration or augmentation is clinically required to address neurodegenerative diseases such as spina bifida, where bladder control and function become severely impaired, as well as in cases of cancer or trauma.[15] Despite the clinical need, very few scaffolds for tissue regeneration have shown promise without culturing cells on the scaffold prior to implantation (cell seeding).[16] Shortcomings of currently used biomaterials are primarily attributed to (1) limited biomechanical suitability for dynamic tissue, (2) batch-to-batch reproducibility of natural biomaterials, (3) pro-inflammatory responses, and (4) inadequate biological activity of synthetic biomaterials that do not support holistic tissue regeneration. Therefore, for bladder tissue engineering as well as the broader tissue engineering and regenerative engineering fields, there is a need for a mechanically durable, cell-free biomaterial with intrinsic bioactivity that can be feasibly and reproducibly manufactured.

Numerous approaches have been pursued to regenerate tissues and organs, yet the potential benefits of electroactive biomaterials remain vastly underutilized. Increased implementation of electroactive biomaterials has shown promise to improve biomaterial bioactivity, even in the absence of external stimulation.[17–19] Conductive polymers are a unique class of organic materials with mixed ionic/electronic conduction that are increasingly being incorporated into biomaterials.[17, 20, 21] Incorporation of conductive polymers into a biological matrix tunes the ionic microenvironment, which mediates fundamental processes of life including cell adhesion, migration, and proliferation.[21–26] Previous studies have documented that conductive polymer-mediated modulation of the ionic environment facilitates more favorable regeneration for nerve, muscle, and epithelial repair, all of which are required for bladder tissue regeneration.[27–31] We thus aim to evaluate whether incorporation of a conductive polymer into a biodegradable citrate-based elastomer will create an ionic electroactive environment that promotes safe and effective bladder regeneration.

Although the biological benefits of incorporating hydrophobic conductive polymers into hydrophilic biomaterials have been well-documented, the lack of integration between the two materials poses significant complications, affecting structural stability and hinder composite conductivity.[32] To address this issue in the context of bladder tissue engineering where mechanical properties are paramount, we developed a phase-compatible functionalization method, wherein the hydrophobic conductive polymer is integrated into a similarly hydrophobic matrix. Although it has previously been demonstrated that modified water-soluble conductive polymers can be reliably incorporated in hydrophilic water-based materials, the synthesis of these water-soluble units is complex, time consuming, and does not financially or logistically lend itself toward commercialization.[33] Conversely, hydrophobic monomer units, such as aniline, pyrrole, or 3,4-ethylenedioxythiophene (EDOT), are widely available yet require a hydrophobic substrate for functionalization. Citrate-based elastomers are a new class of hydrophobic biomaterial with versatile mechanical, chemical, and biological properties that have been recently used for biodegradable medical implants approved by the U.S. Food and Drug Administration.[34] In particular, poly(octamethylene-citrate-co-octanol) (POCO) exhibits mechanical properties under cyclic tension that are suitable to tissues and organs with physically intensive requirements.[35] In previous studies, POCO scaffolds pre-seeded with both CD34 + hematopoietic stem/progenitor cells and mesenchymal stromal cells (MSCs) demonstrated remarkable benefits for bladder tissue regeneration, but the need for pre-seeded cells complicates manufacturability and limits the material's overall translational potential.[36]

Herein, we describe the successful use of a phase-compatible functionalization method to create a biocompatible electroactive and bioactive elastomeric scaffold whereby the conductive polymer poly(3,4-ethylenedioxythiophene) (PEDOT) is incorporated into POCO. We evaluated the safety and efficacy of the PEDOT-POCO scaffolds in a rodent bladder partial cystectomy model and demonstrate that we can restore bladder function to levels that are comparable to those achieved with a cell-seeded POCO scaffold.

## Results

#### In situ coacervation yields stable PEDOT-POCO composites

For preparation of PEDOT-POCO, cured POCO films were first passively infused with a mixture of EDOT and uncured POCO pre-polymer (Fig. 1a). The addition of POCO serves as both a plasticizer and stabilizer in the functionalization of larger PEDOT-POCO films (Fig. 1b). To optimize the addition of POCO in the EDOT solution, EDOT-POCO mixtures with various POCO dilutions were polymerized into PEDOT-POCO nanoparticles and subsequently characterized (Fig. 1c). Initial addition of POCO at a 1:1000 dilution increased PEDOT nanoparticle size, from 560.5 ± 60.8 nm initially to 955.6 ± 32.5 nm, indicative of complexation between these molecules. Further addition of POCO decreased the size of polymerized PEDOT-POCO particles to 736.6 ± 26.6 nm and 588.5 ± 18.8 nm with 1:100 and 1:10 dilution ratios, respectively. With more POCO added to the PEDOT nanoparticles, electrostatic interactions between the molecules are strengthened, resulting in smaller composite particles. The 1:100 POCO:EDOT ratio minimized PEDOT-POCO particle polydispersity, indicating that this dilution optimized molecular interactions and particle homogeneity (**Supplementary Fig. 1**). This dilution ratio was thus selected for further film functionalization.

Polymerization of EDOT-POCO mixtures within the POCO film was performed using an aqueous oxidative solution, which initiates polymerization of the EDOT complexed with POCO. This water-based polymerization approach initiates PEDOT-POCO coacervate within the POCO matrix, driving the formation of nanoparticle-like structures throughout the bulk of the film. The presence of these PEDOT-POCO nanofeatures was visualized and confirmed through scanning electron microscopy (SEM) (Fig. 1d). Nanoparticles, similar to those that were generated *ex situ*, were apparent throughout the PEDOT-POCO composite. Visual inspection of the particulate dimensions revealed features that were comparable in size to those measured *ex situ* (~ 500-700nm).

POCO is rich in carboxyl groups, and we hypothesized that the negative charge of these functional groups contributed to stable, electrostatic hole interactions with the PEDOT backbone.[37] Surface chemistry of PEDOT-POCO films was evaluated and compared to POCO for further characterization of the effects of conductive polymer incorporation. PEDOT-POCO films demonstrated significantly reduced hydrophobicity compared to POCO, measured through contact angle measurements (Fig. 1e). Contact angle for POCO scaffolds was 52.9 ± 8.9 and 36.5 ± 6.9 for PEDOT-POCO. It is feasible to expect that the addition of a hydrophobic filler (PEDOT) to an already hydrophobic matrix (POCO) would increase the composite hydrophobicity, yet in this case we observed the opposite effect in that PEDOT-POCO surfaces were more hydrophilic. This result supports the hypothesis that PEDOT-POCO complexes are driven by electrostatic interactions with carboxyl groups, since those functional groups are dominantly hydrophobic. Composite surface charge was also measured, as it plays a significant role in regulating adhesion and other cellular processes (Fig. 1f).[38, 39] For PEDOT-POCO, surface charge was significantly more positive (-22.1 ± 0.9 mV) than that of the POCO film alone (-29.6 ± 2.7 mV), which can again be attributed to PEDOT sequestering negative charges of the carboxyl groups within POCO (Fig. 1g).

## PEDOT functionalization of POCO maintains desirable mechanical, degradation, and antioxidant properties

POCO has been validated as a promising and effective biomaterial that facilitates tissue function restoration in mechanically intensive applications such as orthopaedic and cardiovascular engineering.[35] We thus set out to maintain the mechanical and biological suitability of POCO when designing PEDOT-POCO films, the end goal being to preserve favorable POCO mechanical, degradation, and antioxidant qualities while enhancing the cellular electronic/ionic microenvironments through PEDOT functionalization. Antioxidant properties of citric acid-based materials are among their most notable advantages for facilitating repair *in vivo*.[40] These potent antioxidant characteristics are owed to the structure of citric acid, and we thus sought to determine whether PEDOT incorporation impaired the inherent antioxidant capabilities of POCO. A DPPH assay was performed to evaluate the free radical scavenging capabilities of PEDOT-POCO and POCO, with polytetrafluoroethylene (PTFE) serving as a negative control (Fig. 2a). PEDOT-POCO exhibited higher free radical scavenging than POCO alone, with the materials exhibiting 23.1 ± 4.8% and 16.0 ± 5.6% scavenging respectively. These results are in line with previous studies documenting free radical scavenging capabilities of conductive polymers including PEDOT.[41–43]

In addition to its antioxidant properties, POCO is an attractive biomaterial for its degradability. Degradability continues to pose a major challenge in the landscape of conductive biomaterial design, as conductive polymers themselves are not typically degradable and may impair the degradability of host matrices.[18, 44, 45] An accelerated degradation assay was performed by incubating POCO and PEDOT-POCO in phosphate-buffered saline (PBS) at 70 C to evaluate whether the material was capable of degradation, despite the PEDOT functionalization. We found that PEDOT-POCO bulk, specifically the POCO-based constituents, did dissolve while precipitated PEDOT nanoparticles remained, the sizes of which were comparable to those observed in the SEM images (Fig. 2b). The solution the material was degraded in was pipetted out from the solid PEDOT particles and analyzed through FTIR **(Supplementary Fig. 2)**. The FTIR spectra of the leached solution showed no notable differences compared to the reference PBS spectra, indicating that the remaining PEDOT is the only significant by-product of film degradation, which precipitates out of the solution. After verifying the maintenance of biodegradability, we screened PEDOT-POCO for cytotoxicity via alamarBlue cell activity assay. Human bone marrow-derived mesenchymal stromal cells (MSCs) were seeded on fibronectin-coated materials. No differences in cell viability were apparent with cells seeded on PEDOT-POCO compared to POCO alone (Fig. 2c).

Finally, we verified that phase-compatible PEDOT functionalization sustained favorable POCO mechanics and elastomeric behavior. In several biological applications, such as bladder or cardiac engineering, material robustness and low modulus are vital towards the success of the application. Few stretchable conductive materials demonstrate both elasticity and ~ kPa modulus. Tensile testing as well as cyclic biaxial testing was performed (Fig. 2d-f **Supplementary Fig. 3)**. Modulus, elongation, and elastic behavior of PEDOT-POCO were all comparable to that of the pristine POCO, with Young's modulus of PEDOT-POCO measured as 630 ± 188 kPa, whereas the modulus of POCO is 445 ± 87 kPa. The increased stiffness was not unexpected, as conductive polymers have been well-documented to increase material modulus, yet it is not expected that this magnitude of difference in modulus will bear impact regarding the material's capability to facilitate regeneration. For bladder regeneration, the complex mechanical loads the tissue is subject to in conjunction with a diversity of tissue types require candidate materials to satisfy a stringent set of standards. We thus have demonstrated that PEDOT-POCO performs comparably to POCO as evaluated against key metrics including antioxidant activity, biocompatibility, and mechanical suitability.

## PEDOT incorporation into POCO differentially affects ionic and electronic microenvironments

The mixed ionic/electronic conductivity of conducting polymers, including PEDOT, is widely cited as a quality that functionally differentiates these materials from others.[21] For biological applications, ionic conduction is particularly attractive considering the vital influence of ions in life processes. Regardless of this recognized benefit, the specific changes in ionic microenvironment following conductive polymer incorporation remains largely uncharacterized. During material preparation, films are subjected to a leaching protocol to ensure that unreacted reagents, such as oxidant or polymer, are removed. Prior to functionalization with PEDOT, POCO films are leached in solutions with gradually increasing osmolarity, with the final step being cell media. After PEDOT functionalization, PEDOT-POCO is again leached in cell media. This leaching protocol establishes a specific ionic microenvironment that varies between POCO and PEDOT-POCO, despite the primary source of ions (cell media) being consistent. To better understand how ionic content of the films was altered by PEDOT incorporation, energy dispersive x-ray spectroscopy (EDS) was performed on film cross-sections. Levels of magnesium, sodium, chlorine, and calcium were examined in POCO and PEDOT-POCO (Fig. 3a-b). We found that compared to POCO, PEDOT-POCO films contained lower levels of magnesium and sodium but higher levels of chlorine and calcium. We hypothesize that POCO retains higher levels of small, monovalent ions due to trapping effects of the negatively charged carboxyl groups, which are unencumbered in the pristine POCO case (Fig. 3c). Chlorine levels were also dramatically elevated in PEDOT-POCO as compared to POCO likely due to interactions of negative chlorine ions and the positively charged PEDOT backbone.

After characterizing differences in the ionic makeup of the films after PEDOT incorporation, the electrochemical properties of the films were characterized using a two electrode-setup with both cyclic voltammetry (CV) and electrochemical impedance spectroscopy (EIS) (Fig. 3d-f). POCO CV demonstrated oxidative and reduction peaks that are characteristic of citric acid and its scavenging capabilities.[46] As expected, PEDOT incorporation elevated conductivity, evidenced by both CV and EIS results. PEDOT-POCO conductivity is estimated to be ~ 0.3 S/m, based on 2-point resistance probing via CV. After validating that PEDOT-POCO bolstered passive film conductivity, we sought to evaluate the electronic performance of the material under mechanical stress, which it would experience *in vivo* (Fig. 3g). To assess electromechanical durability of the films, chronoamperometry was performed during tensile and biaxial testing (Fig. 3h,i). During tensile testing to 30% strain, PEDOT-POCO demonstrated ~ 5-times higher sensitivity to deformation than the POCO films alone, the resistance of which did not change remarkably after a single deformation up to 30% strain. Changes in resistance were also measured throughout cyclic testing. During POCO biaxial testing, film resistance increased outside the measurable range prior to 500 deformation cycles. In contrast, resistance of PEDOT-POCO films was lower than that of POCO throughout the duration of testing. We have thus demonstrated PEDOT-POCO is mechanically and electronically robust for sustaining repeated cyclic deformation without undergoing significant damage to the film integrity or electrochemical performance.

## PEDOT-POCO enables functional bladder recovery that is comparable to cell-seeded scaffolds

After performing *in vitro* characterization on the PEDOT-POCO films and verifying their preliminary suitability for bladder repair applications, we evaluated efficacy for promoting regeneration in a rat bladder augmentation model (Fig. 4a). Since acellular scaffolds have historically demonstrated limited success for facilitating bladder regeneration as efficiently as their cell-seeded counterparts, we investigated 3 experimental groups: POCO, cell-seed POCO, and PEDOT-POCO. By comparing the PEDOT-POCO scaffold to a cell-seeded, non-conductive scaffold, we benchmark the regenerative potential of PEDOT-POCO scaffolds to a condition that is considered the optimal approach to maximize regenerated tissue quality.[16] With cell-seeded scaffolds, human MSCs were seeded in conjunction with CD34 + hematopoietic progenitor cells as previously described.[47] This approach has demonstrated significant potential to facilitate functional bladder regeneration, including when used in conjunction with citrate-based biomaterials such as POCO.[36, 47]

Bladder regeneration was assessed at 4 weeks post-augmentation. Urodynamics measurements are the key clinical indicator for bladder performance and were thus the assessment weighed most heavily when evaluating the performance of regenerated bladder tissue (Fig. 4b-c). Compliance and void frequency were derived from these measurements while capacity, another vital indicator of bladder performance, was measured directly **(Figure d-f)**. Increased void frequency is associated with overactive bladder conditions and has been associated with dysregulated bladder sensation.[48, 49] Assessment of void frequency in POCO, cell-seeded POCO, and PEDOT-POCO groups showed that both cell-seeded POCO and PEDOT-POCO reduced void frequency to comparable degrees, which was significantly lower than the POCO only group. In addition to void frequency, compliance and capacity were evaluated, with the former typically serving as the clinical indicator of limited bladder performance requiring intervention. Similarly to void frequency, both cell-seeded POCO and PEDOT-POCO comparably improved bladder compliance and capacity while significantly exceeding the tissue quality achieved by animals augmented using POCO only.

Taking these results together, we have demonstrated the potential for a purely synthetic biodegradable biomaterial, in this case PEDOT-POCO, to perform comparably to a cell-seeded scaffold in facilitating functional bladder regeneration. The use of cell-seeded materials has demonstrated significant benefits throughout the regenerative engineering field, yet we show here that conductive polymer-elastomers have the potential to match the quality of regenerated tissue while reducing cost and complexity of manufacturing such materials.

## PEDOT-POCO supported the formation of various tissue types integral to bladder function

The capability for conductive polymers to induce differentiation and regeneration of tissue types including nerve, muscle, and epithelium has been well-documented in several previous studies.[17, 25, 50, 51] However, their use for the regeneration of multiple tissue types within an organ, such as the bladder where regeneration of distinct cell lineages is required for restoration of holistic function has not been reported. Furthermore, after observing that PEDOT-POCO improves bladder function comparable to the cell-seeded POCO, we examined the regenerated organ more closely to determine how the conductive polymer influenced regeneration of specific tissues subtypes. A variety of histological analyses were performed to determine the quality of regenerated tissue subtypes. Trichrome staining was used to quantify urothelium, or epithelial bladder lining, thickness, muscle to collagen ratio, and blood vessel size (Fig. 5a). Nerve regeneration was quantified by immunofluorescence staining of β-III tubulin **(Supplementary Fig. 4)**.

The urothelium is a critical anatomical feature that enables the bladder to safely interface with the nitrogenous waste it contains.[52] An inability for scaffolds to withstand this harsh environment has been one longstanding limitation in the design of biomaterials for bladder regeneration.[16] Furthermore, the success of a material depends heftily on its capability to facilitate urothelium regrowth (Fig. 5b). PEDOT-POCO promoted the growth of thicker urothelium (44.5 ± 24.6 μm), on average, than POCO alone (19.7 ± 12.2 μm), and was statistically indeterminate from cell-seeded POCO (48.4 ± 6.5 μm). Despite the statistically comparable performance, cell-seeded POCO regenerated thicker urothelium on average when compared to the PEDOT-POCO scaffold.

In addition to urothelium regeneration, muscle to collagen ratio of regenerated bladder tissue was analyzed. Excessive collagen production can be associated with inflammation and overall improper tissue regeneration. Smooth muscle tissue, however, is a critical constituent of the bladder wall and is vital for facilitating passive low-pressure bladder filling as well as subsequent voiding, or bladder emptying. Furthermore, the muscle:collagen ratio is quantified by analyzing the levels of red (muscle) to the levels of blue (collagen) throughout the trichrome images (Fig. 5c). Image quantification confirms this qualitative observation, with the PEDOT-POCO tissue having a significantly higher muscle:collagen ratio than the POCO alone, with values of 0.48 ± 0.18 and 0.22 ± 0.09 respectively. Native muscle:collagen ratio is typically approximately 0.58, demonstrating that PEDOT-POCO recapitulates tissue organization that is closer to the initial physiological state.

Vasculature is another vital component that is vital for bladder regeneration. Conductive polymers have remained largely unstudied in the context of vasculature regeneration, making this study among the first to examine their potential to promote new blood vessel infiltration and development (Fig. 5d).[53] Area of the average regenerated vessel in PEDOT-POCO bladders was ~ 30% larger than those in POCO only bladders yet ~ 25% smaller than those in cell-seeded POCO bladders. PEDOT-POCO's capability to increase regenerated vessel size compared to the POCO-only group is promising and points toward the capabilities for conductive polymers to facilitate vasculature regeneration in future applications.

Finally, we examined the average length of the peripheral nerve elements regenerated throughout the bladder tissue (Fig. 5e). Nervous system regeneration is one of the most popular applications that electronic materials are currently implemented towards, in part due to the widely-recognized electrogenic nature of this tissue type. As was observed with urothelium, smooth muscle, and vasculature, PEDOT-POCO significantly improved the average length of the nerves in regenerated bladder tissue compared to POCO alone. The cell-seeded POCO again performed the best among all of these conditions promoting growth of nerves that were on average 32.7 ± 5.2 μm long compared to 26.5 ± 5.1 μm with PEDOT-POCO and 20.2 ± 2.6 μm with POCO alone .

## Discussion

A variety of materials have been explored for bladder engineering including acellular natural materials, synthetic polymers, and naturally derived polymers.[54] Submucosal intestinal submucosa (SIS) and bladder acellular matrix (BAM) are among the most widely explored scaffolds for bladder regeneration, yet these scaffolds present poor mechanical properties for this application.[55] Without the inclusion of cells, associated complications ensue such as fibrosis and stone formation, none of which were observed in our study.[54, 56, 57 58] A variety of cell types have been investigated for bladder regeneration including urothelial cells, smooth muscle cells, adipose-derived stem cells, and MSCs.[47, 59, 60] Results from these studies support the need for exogenous cells seeded on scaffolds for the recapitulation of bladder function and have thus served as a beacon guiding the field's standards. Despite the clinical potential for this approach, cell-seeded scaffolds do present with regulatory, manufacturing, and adoption barriers to widespread commercialization. From a regulatory point of view, the inclusion of cells on a scaffold is likely considered a combination product, requiring extensive clinical trials to demonstrate safety and efficacy. Cell manufacturing is expensive and difficult to reliably implement on a large scale. Regarding clinical adoption, cell-seeded scaffolds require special controls for transport and storage and in the case of an autologous cell source, require operations for both cell/tissue harvesting and scaffold implantation.[16] Offering surgeons a scaffold that has higher processability, manufacturability, and simplicity than the cell-seeded scaffold alternative is expected to simplify the translational pathway for bladder tissue engineering.

When evaluated in a bladder augmentation model, the PEDOT-POCO scaffold, in the absence of seeded cells, demonstrated the capability to regenerate bladder tissue and restore organ function. The PEDOT-

POCO scaffold properties achieved, including low modulus, elasticity, stretchability, electrical conductivity, and bulk degradability, enabled the first implementation of an electroactive scaffold in a bladder augmentation model. Use of conductive polymers in tissue engineering has been limited due to biocompatibility and mechanical property mismatch challenges for the tissue or organ regeneration application. Other studies have sought different approaches for improving the mechanical properties of PEDOT-based materials.[32, 61–63] Previous research has complexed the popular PEDOT:polystyrene sulfonate (PSS) dispersion with rubber, polyurethane, or other co-polymers.[61, 64, 65] While such composites show remarkable conductivity and stretchability, the modulus is typically higher than 1 MPa, and cytotoxicity often not reported. In our study, we introduce a novel biocompatible electroactive elastomer with first-of-its-kind structural stability and bulk degradability.

In this study, we also demonstrate the capability for conductive materials to promote regeneration intrinsically, without external stimulation. Many regenerative engineering studies implement electroactive materials in conjunction with external electronic stimulation such as applied currents or potentials to modulate cellular processes.[17, 50, 66] Yet, active stimulation regimes mask the passive influence of the electroactive material, which we have demonstrated here to be independently effective. Electroactive materials have been predominantly examined for nerve regeneration.[67–69] They have also shown promise for their ability to facilitate epidermal tissue and muscle regeneration.[51, 67, 70, 71] Given that PEDOT-POCO scaffolds facilitated the simultaneous restoration of multiple tissue types, including urothelium, smooth muscle, nerve, and blood vessels, we demonstrate the feasibility of electroactive polymer or scaffold systems to recapitulate the anatomical complexities of other organs.

One mechanism conductive polymers passively modulate biological activity is through rearranging the ionic microenvironment.[72] Ions are responsible for regulating various cellular processes and can influence gene expression directly.[73] We incorporated PEDOT within POCO films to create scaffolds that modulate cellular electronic and ionic microenvironments, bolstering the scaffold's regenerative potential.[21, 74] Conductive polymers can drive increased $Ca^{2+}$ signaling as a key factor that regulates regenerative processes.[22, 75, 76] PEDOT-POCO scaffolds facilitate higher passive $Ca^{2+}$ concentrations than POCO scaffolds. The specific biophysical relationship between conductive polymers and upregulated $Ca^{2+}$ induction has yet to be elucidated, although there is evidence pointing to membrane depolarization as a key transducer in initiating differential cellular ion fluxes and influencing signaling.[77–80] The exact mechanism and source of differences in ionic microenvironments are thus under continued investigation. Enhanced understanding of material-regulated ionic dynamics would be instrumental in advancing the design of more effective biological scaffolds.

Future work should aim to investigate longer-term outcomes and mechanisms associated with PEDOT-POCO degradation *in vivo*. Preliminary examination of inflammatory cell populations revealed comparable M1:M2 ratios with both POCO and PEDOT-POCO bladders **(Supplementary Fig. 5)**. This result is highly promising as it suggests that the incorporation of PEDOT into the POCO matrix does not cause severe acute inflammatory responses that could be detrimental. While the short-term examination of immune response is promising, future investigation is warranted to better understand the physiological processing and longer-term risks of PEDOT-POCO as it degrades. One limitation of the present study is the use of an immunocompromised small animal model due to the need to include scaffolds seeded with human cells for comparison. To further screen the safety and efficacy of the PEDOT-POCO scaffold, its regenerative potential should be evaluated in a large animal model, which will be the focus of future studies.

### Methods

## POCO polymer and scaffold/film synthesis

POCO was synthesized according to previously published methods.[35] Briefly, citric acid, 1,8-octanediol, and octanol were stirred and melted at 160  C with liquid nitrogen flow until the mixture turned transparent, approximately 15 minutes, then the temperature was lowered to 145 C. Stir speed started at 500 rpm, and stir speed was subsequently reduced until the stir bar could not spin smoothly in the solution. After synthesis, the polymer was purified three times by dissolving in ethanol and precipitating out in MilliQ water. After purification, the pre-polymer was then diluted to 40 wt% in ethanol for film synthesis.

Glass slides were prepped for POCO films by rinsing in DI water and drying with nitrogen. Polyvinyl alcohol (PVA) solution was prepared at a concentration of 50 mg $mL^{-1}$ in MilliQ water, and 2 mL of PVA was pipetted onto each glass slide and then cured at 65 C for 1.5-2 hours until slides were dry. 2 mL of POCO was pipetted onto the PVA slides and left out at room temperature overnight to allow for excess solvent evaporation. After the overnight evaporation, films were cured at 65 C for 4 days.

POCO films were then incubated in DI water overnight to dissolve the PVA and lift the POCO from the glass slide. Subsequent leaching of the film was performed to remove unreacted carboxyl groups from the film. Films were leached in 20% ethanol in PBS with 1% penicillin/streptomycin for 24 hours at 37   C, followed by PBS with 1% penicillin/streptomycin for 24 hours at 37   C. Films were then leached in low glucose DMEM with 1% penicillin/streptomycin for 2 hours, followed by a brief rinse in DI water, and low glucose DMEM was readded to the film and leached overnight. Finally, the film was leached in high glucose DMEM with 10% FBS and 1% penicillin/streptomycin. At this step, films were ready for PEDOT functionalization. To sterilize the POCO films for downstream use, they were cut into desired shape, incubated in 70% ethanol for 20 minutes, and leached overnight at 37 C in high glucose DMEM with 10% FBS and 1% penicillin/streptomycin to remove excess ethanol remaining on the film.

In situ *PEDOT-POCO oxidative polymerization*

After complete leaching, POCO films were functionalized with PEDOT first by incubating the films in EDOT with a 1:100 dilution of POCO pre-polymer for 72 hr at room temperature. Films were then moved to a polymerization solution containing 28.5% EDOT, 14.2% 1.24 M ammonium persulfate, and 57.3% phytic acid, and vigorously mixed at 4 C overnight. After polymerization, films were incubated in 70% ethanol for 15 minutes and rinsed in high glucose DMEM with 10% FBS and 1% penicillin/streptomycin until the solution stopped changing color, to remove excess polymerization solution. Films were then incubated at room temperature in high glucose DMEM with 10% FBS and 1% penicillin/streptomycin for 24 hours, and the next day, leaching solution was replaced and films were transferred to 37 C and leaching was continued for 24 hours.

## Fourier Transform Infrared (FT-IR) Spectroscopy

Fourier transform infrared (FT-IR) spectroscopy was performed on a Nicolet iS50 spectrometer using attenuated total reflection (ATR) spectroscopy to analyze the composition of degraded PEDOT-POCO leach solution. Spectra were collected with OMNIC Software.

## Zeta Potential Measurements

Zeta potential and particle size measurements were evaluated on a Malvern Zeta Sizer. For measurements of PEDOT particle size with POCO dilutions, PEDOT polymerization solution was prepared as described in the PEDOT-POCO in situ polymerization section and POCO was added at either 1:1000, 1:100, or 1:10 dilution levels. The resulting solution was vigorously mixed overnight at 4 C. The polymerized solution was then diluted at 1:100 in ethanol for particle size measurements. To analyze film surface charge, PEDOT-POCO and POCO were frozen in liquid nitrogen then samples were pulverized, suspended in PBS, and filtered through a 70 µm mesh. Surface charge was quantified in triplicates and three different samples of solution were evaluated.

## Scanning Electron Microscopy (SEM) & energy dispersive X-ray spectroscopy (EDS)

Samples were dried overnight at room temperature and then coated in carbon with a Denton III Desk Sputter Coater. EDS was performed on a Hitachi SU8030 to visualize the distribution of sulfur throughout the cross-section of the PEDOT-POCO film. Data was collected and processed using Aztec software.

## Free Radical Scavenging

Free radical scavenging was measured to assess PEDOT-POCO antioxidant activity. Samples were incubated for 24 hours at 37  C in 200 µM DPPH dissolved in ethanol. PTFE was used as a negative control, and ascorbic acid was used as a positive control. The positive control measurement was subtracted from all samples and normalized to the blank. Absorbance was measured at 320 nm to minimize precipitate background effects. All samples were measured in triplicate.

## Contact Angle Measurements

Sample contact angles were measured with an Ossila Contact Angle Goniometer and measurements were collected with the corresponding Ossila software. Reports of contact angle were logged and measured in triplicate.

### Electrochemical Measurements

EIS and CV were conducted using a two-probe set up with reference and working electrodes. The counter electrode was shorted to the reference. Pogo pins 4 mm apart were used to conduct measurements. EIS was executed from 0.1 to $10^6$ Hz. CVs were performed by cycling from − 0.6 to 0.6 V 5 times. Measurements were collected with a Palmsens 4 and analyzed in the PS Trace software.

### Mechanical & Electromechancial Testing

Tensile testing was performed using an Instron and data was collected via the Bluehill Universal software. All experiments were conducted in triplicate. Young's modulus was calculated by measuring the slope in the linear region of the tensile curve. Strips were cut into 5 x 1 mm rectangles, and a strain rate of 15 mm $min^{-1}$ was utilized for testing. For biaxial testing, a maximum strain of 30% was applied and material was elongated for 1000 cycles. To collect electromechanical measurements, copper tape was attached to the Instron grips which served as contact points for Palmsens 4 alligator clips. Chronoamperometry was then performed to measure changes in potential with the application of a constant current. Electromechanical data was collected using the PS Trace software.

### Animal Surgeries & Urodynamics Studies (UDS)

Charles River 10-week old athymic rats weighing approximately 200 g underwent bladder augmentation procedures as previously described (n = 6 per group).[47, 81] For POCO and PEDOT-POCO augmentations, all animals were female. For cell-seeded POCO, 4 females and 2 males were used. Briefly, rats were anesthetized and the bladder dome was excised through a 1-cm midline abdominal incision. Rats underwent a 50–60% partial cystectomy and the appropriate scaffold was sutured around the bladder defect using 7 − 0 polyglactin suture. An omental wrap was then sutured around the scaffold-augmented bladder. The rat abdomen was then closed with 5 − 0 ethibound and finally with 9-mm autoclips at the skin surface.

UDS were performed prior to augmentation procedures and 4-weeks post-augmentation. Animals were anesthetized and catheterized with the abdomen closed to measure capacity. The bladder was manually massaged to facilitate emptying. A 1 mL syringe was then used to fill the bladder until a voiding event was observed. The total volume infused into the bladder at the time of voiding was measured to determine bladder capacity. After measuring capacity, the rat abdomen was opened with a 1 cm incision and a 20 gauge cannula (Becton Dickinson) was inserted into the bladder wall to assess urodynamics. The needle was secured to a transducer and syringe pump for consistent fluid injection. The syringe pump was set to a flow rate of 150 µL $min^{-1}$. Recordings of pressure over time were collected in a custom LabView program that included at least 5 voiding events. From this data, void frequency and compliance were analyzed. Compliance is calculated as the fraction of time during voiding in which the bladder pressure is less than 20 $cmH_2O$.[82]

## Histological Assessments

Animals were euthanized 4 weeks post-augmentation and bladder tissue was collected and fixed in 10% buffered formalin (Fisher Scientific) overnight. After fixation, samples were gradually dehydrated and embedded in paraffin. Sectioning was performed in 5 µm slices. Samples were then deparaffinized accordingly through well-established protocols for either trichrome or immunofluorescence staining. Portions of the regenerated tissue were then imaged using a 40x objective on an Eclipse Ti2 Nikon microscope. For each histological assessment, 3 images were evaluated per tissue section. Urothelium thickness was measured in at least 3 unique locale per image from the trichrome-stained slides in ImageJ. For each animal, all measurements were averaged and taken to represent the average urothelium thickness. Muscle:collagen ratio and vasculature quantification was determined as previously described.[81]

Immunofluorescence was performed to evaluate peripheral nerve regeneration as well as macrophage (M1:M2) ratios. To evaluate bladder tissue peripheral nerve regeneration, an anti-β-III tubulin antibody (Biolegend) was utilized to stain bladder tissue at a 1:250 dilution for regenerating peripheral nerves. Peripheral nerve length was measured in ImageJ. For a signal to be measured as peripheral nerve, at least two distinct nuclei were required to be considered an element. This ensured that only elements within the appropriate plane were quantified. For M1:M2 quantification, an anti-CD86 antibody (Thermo, 1:100 dilution) was used to stain for M1 macrophages and an anti-CD163 antibody (Thermo, 1:200 dilution) was used to stain for M2 macrophages. Blood vessels were removed from the images due to their autofluorescence potentially confounding quantification, and cell area was measured using the Analyze Particles function in ImageJ.

## Statistical Analysis

Results reported in the text are shown as mean ± standard deviation. Graphs show data set quartiles, with the medians shown as “X” and the mean being indicated as a solid line within the box. Statistical significance was determined using a two-tailed t-test where $p < 0.05$ was considered statistically significant. Significance was indicated with the following indications: $^*p < 0.05$, $^{**}p < 0.01$, $^{***}p < 0.001$.

### Declarations

## Acknowledgments

This work was primarily supported by a National Institutes of Health awards (R01EB026572 (AKS and GAA) and R01DK109539 (AKS). RK was supported in part by the National Institutes of Health Training Grant (Grant No. T32GM008449) through Northwestern University’s Biotechnology Training Program. RK gratefully acknowledges support from the Ryan Fellowship and the International Institute for Nanotechnology at Northwestern University. This work made use of the EPIC facility of Northwestern University’s NUANCE Center, which has received support from the SHyNE Resource (Grant No. NSF

ECCS2025633), the International Institute for Nanotechnology, and Northwestern's MRSEC program (Grant No. NSF DMR-1720139).

### Figures

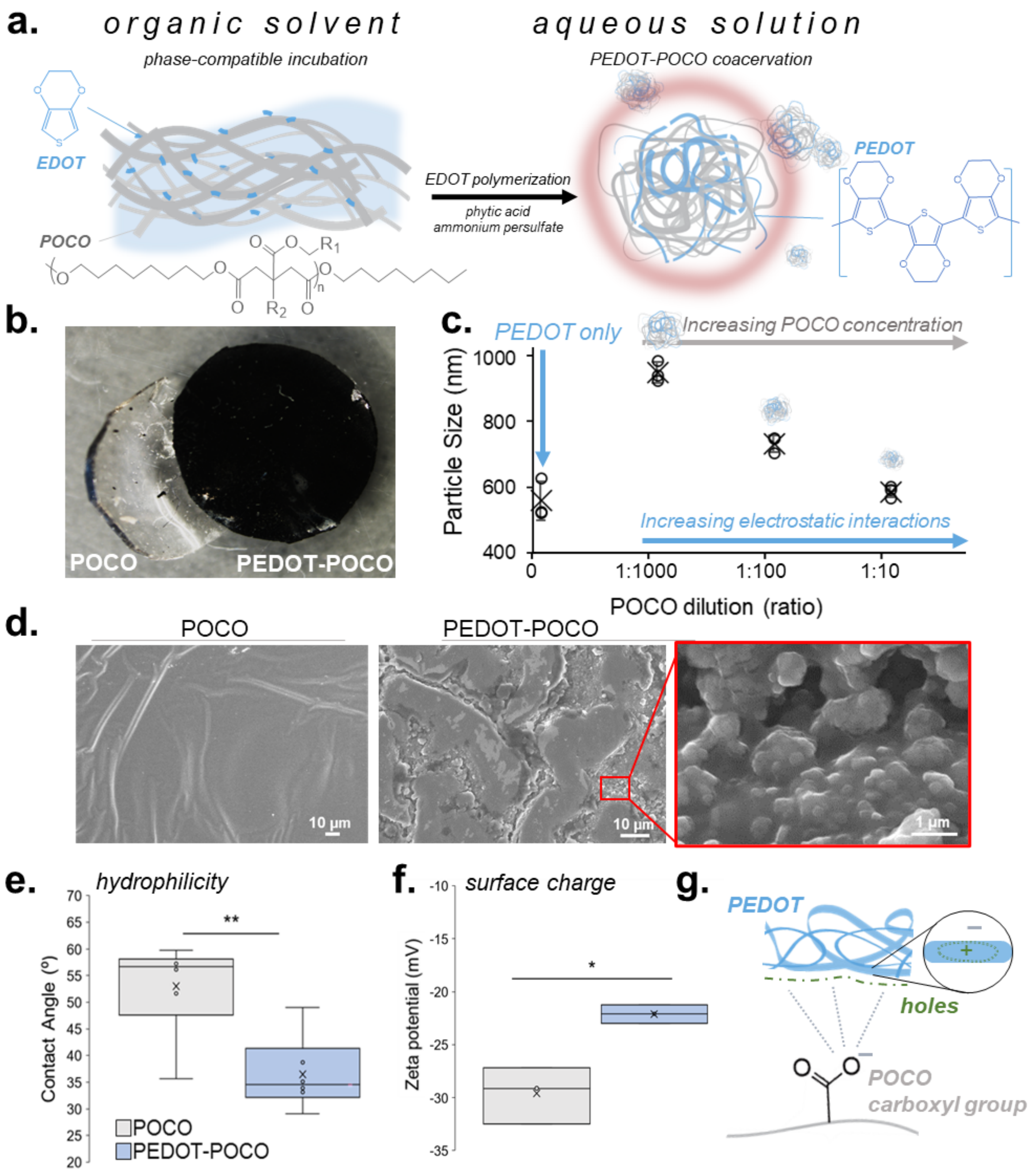

### Figure 1

PEDOT coacervate facilitated by PEDOT-POCO electrostatic interactions (a) Schematic depicting PEDOT-POCO (R1, R2 represent polymer chains) functionalization and proceeding coacervation after oxidation in aqueous environment to obtain (b) functionalized PEDOT-POCO films. (c) PEDOT-POCO particle size characterization was utilized to optimize final functionalization conditions (n=3 runs/solution). The circles indicate individual data points and X marks sample mean. (e) Contact angle measurements (n=6) and (f) zeta potential measurements (n=3 scaffolds, n=3 runs per scaffold) were performed to characterize PEDOT-POCO surface chemistry. (g) The results of these assays point to PEDOT-POCO complexation (* indicates p<0.05, ** indicates p<0.01).

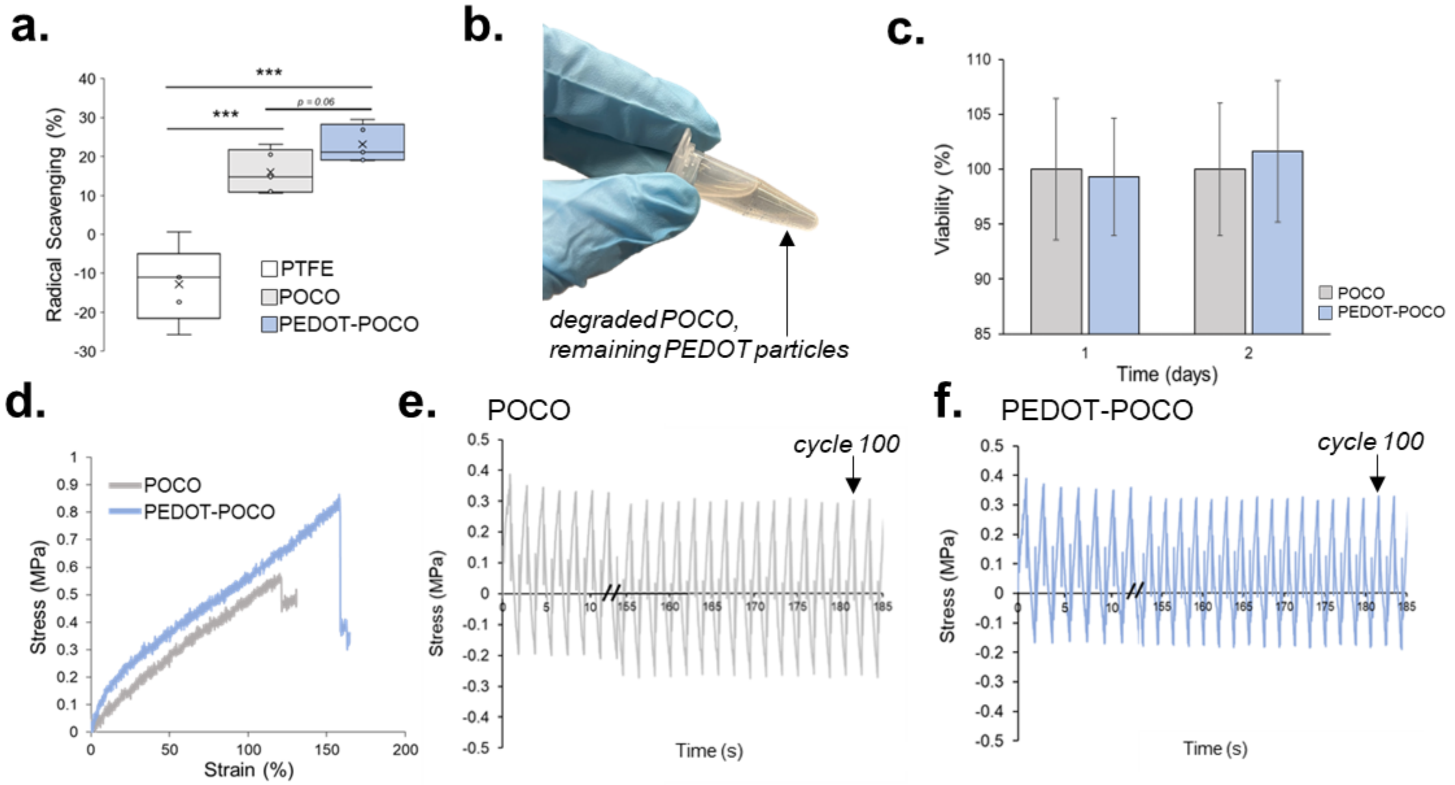

### Figure 2

PEDOT-POCO preserves favorable POCO characteristics **(a)** PEDOT functionalization bolsters antioxidant activity of POCO, as measured through a free radical scavenging DPPH assay (n=5). **(b)** As PEDOT-POCO degrades overtime, PEDOT nanoparticles are remaining while the rest of the scaffold has dissolved. **(c)** Cell viability and **(d)** mechanical properties of PEDOT-POCO are comparable to that of POCO alone (n=3). After multiple cycles **(e)** POCO and **(f)** PEDOT-POCO demonstrate similar elasticity (* indicates p<0.05, ** indicates p<0.01, *** indicates p<0.001).

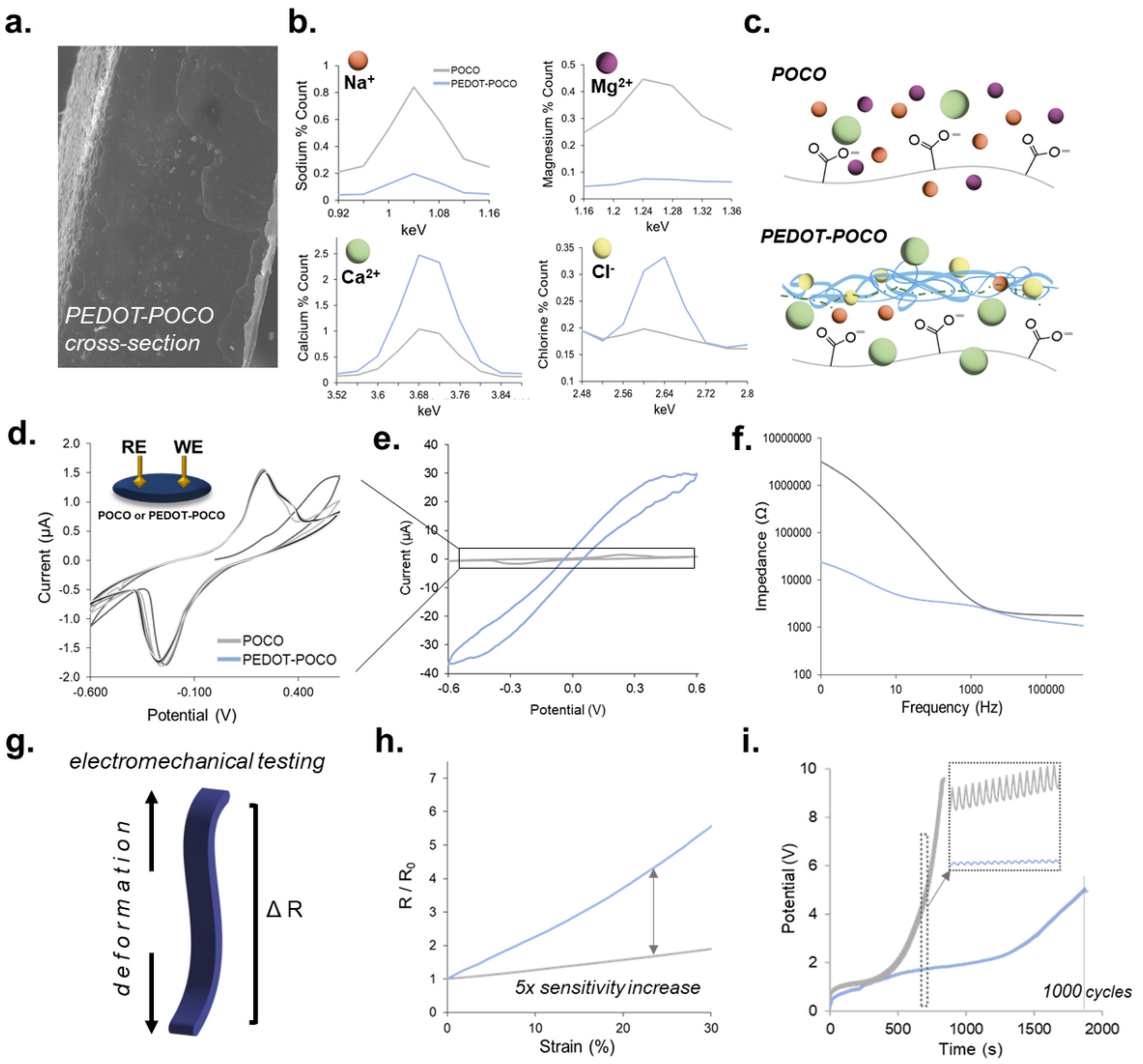

### Figure 3

PEDOT incorporation modifies ionic and electronic properties **(a)** SEM of PEDOT-POCO cross-section used for **(b)** elemental analysis via EDS to determine differences in **(c)** ionic microenvironments. Representative electrochemical analyses of **(d)** POCO and **(e)** PEDOT-POCO via CV and **(f)** EIS revealed an increased conductivity, and decreased impedance at low frequencies with conductive polymer incorporation. **(g)** Electromechanical testing of films showed that PEDOT-POCO exhibited **(h)** higher sensitivity to deformation and **(i)** measurable potentials during cyclic testing (n=3).

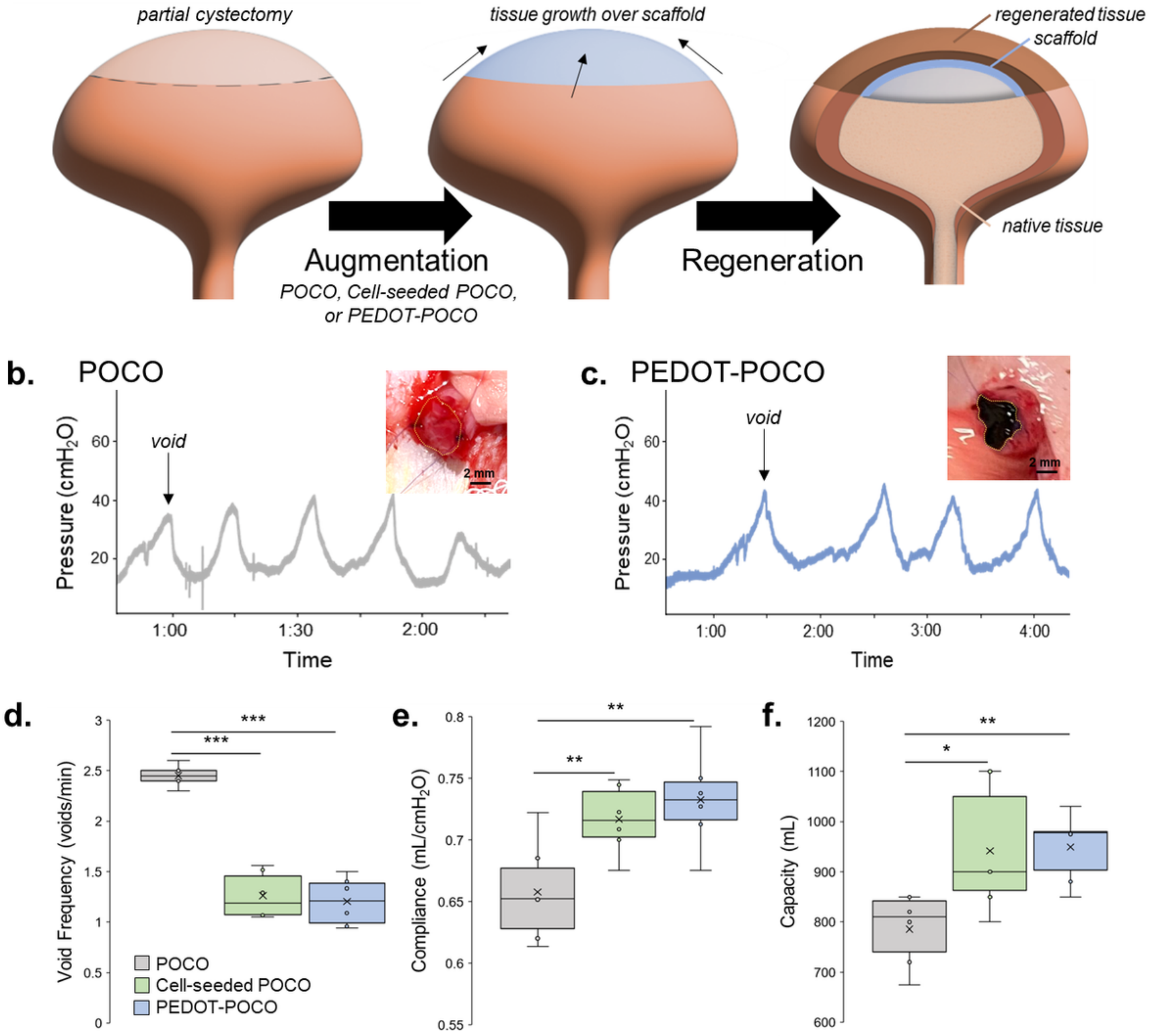

**Figure 4**

Urodynamics assessment of functional bladder regeneration **(a)** Schematic representation of bladder augmentation. **(b, c)** Representative urodynamics tracings of POCO and PEDOT-POCO modified bladders are shown 4-weeks post-augmentation. Insets show images of the bladder at the time of surgery. Functional bladder assessment was evaluated using **(d)** void frequency, **(e)** compliance, and **(f)** capacity as key metrics (n=6 animals per group) (*p<0.05, **p<0.01, ***p<0.001).

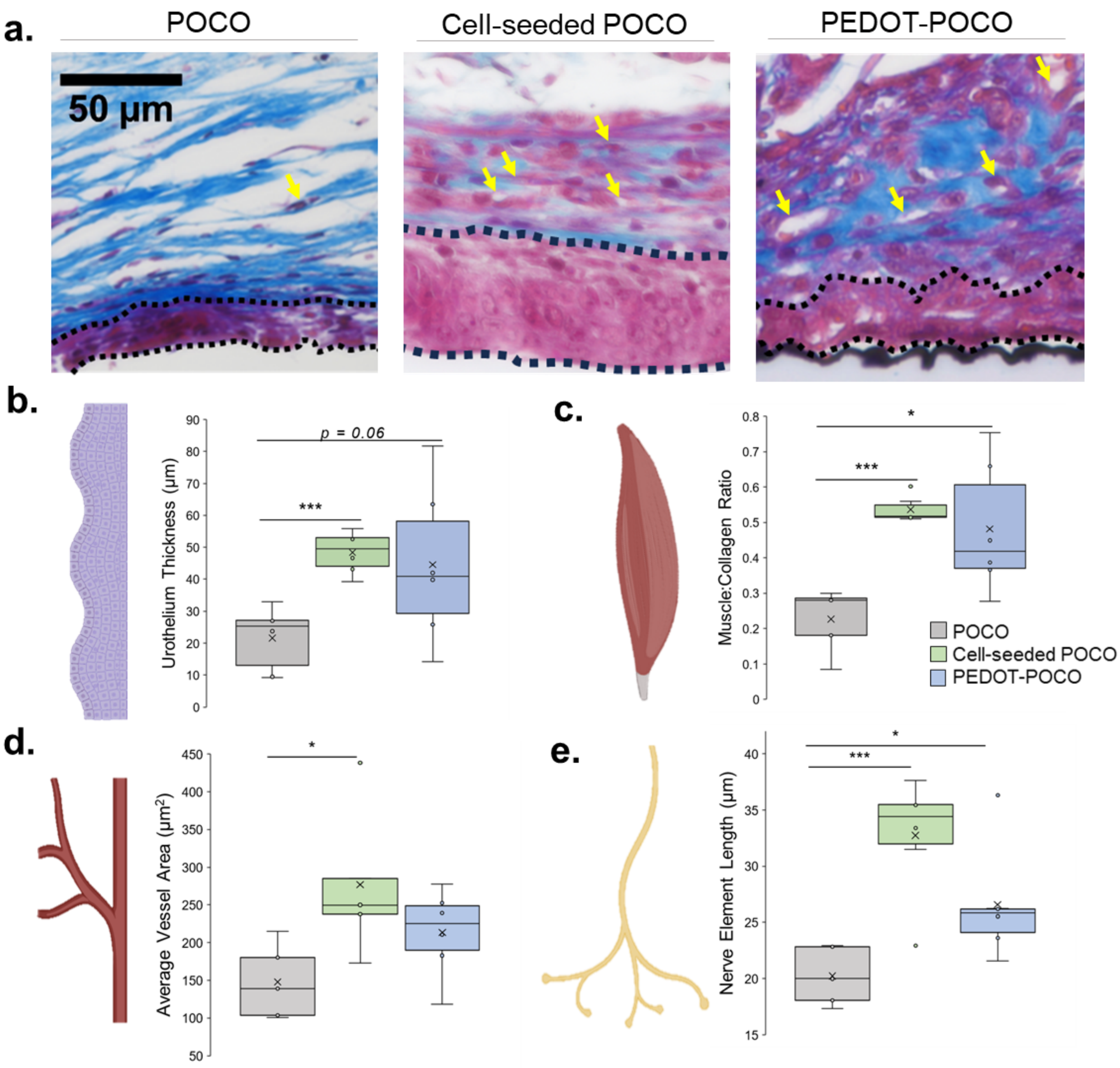

**Figure 5**

PEDOT-POCO improves regeneration in a variety of tissue types compared to POCO alone **(a)** Trichrome staining (red = cytoplasm, blue = collagen) was used to quantify **(b)** urothelium thickness (shown with black dotted lines), **(c)** muscle:collagen ratio and **(d)** vasculature regeneration (examples shown with yellow arrows). **(e)** Regeneration of peripheral nerve elements was analyzed through immunohistology staining of β-III tubulin (images in Supplementary Information) (n=5-6 animals per group) (* indicates $p<0.05$, ** indicates $p<0.01$, *** indicates $p<0.001$).

## Supplementary Files

This is a list of supplementary files associated with this preprint. Click to download.

- SupplementaryInformation.docx
